# Prenatal Diagnosis of Skeletal Dysplasia and Review of the Literature

**DOI:** 10.1155/2021/9940063

**Published:** 2021-04-13

**Authors:** Bashiru Babatunde Jimah, Teresa Aba Mensah, Kofi Ulzen-Appiah, Benjamin Dabo Sarkodie, Dorothea Akosua Anim, Emmanuella Amoako, Evelyn Antwiwaa Gyamfi

**Affiliations:** ^1^Department of Medical Imaging, University of Cape Coast, School of Medical Sciences, Ghana; ^2^Department of Obstetrics and Gynecology, Cape Coast Teaching Hospital, Ghana; ^3^Department of Pathology, Cape Coast Teaching Hospital, Cape Coast, Ghana; ^4^University of Ghana, School of Medical Sciences, Accra, Ghana; ^5^Korle Bu Teaching Hospital, Department of Radiology, Accra, Ghana; ^6^Department of Paediatrics and Child Health, Cape Coast Teaching Hospital, Ghana; ^7^Department of Sonography, RAAJ Specialist Scan, Cape Coast, Ghana

## Abstract

**Introduction:**

Obstetric ultrasonography is routinely used to screen for fetal anomalies. Thanatophoric dysplasia (TD) is one of the common though rare lethal skeletal dysplasia, detected during routine ultrasound scan. TD is caused by a mutation in FGFR3 gene. Characteristic features include shortening of limbs, macrocephaly and platyspondyly. In our local setting, it is common to miss the diagnosis in the early scans due to lack of expertise of the sonographers. To the best of our knowledge, this is the first publication from Ghana. *Case Presentation*. We present the case of a 33-year-old woman who was referred to the facility on account of ultrasound scan report suggestive of thanatophoric dysplasia type 1 at 34 weeks of a female baby. The diagnosis was not made despite the mother being a regular antenatal attendant, until a fifth scan done at 34 weeks reported features suggestive of thanatophoric dysplasia. The ultrasound scan features included a biparietal diameter of 37weeks, femur length—24weeks, narrowed thoracic cage with hypoplastic lungs and short ribs. The liquor volume was increased with amniotic fluid index (AFI) of 38.4 cm. The femur, tibia, fibula, humerus, ulna, and radius were shortened (micromelia). The diagnosis of thanatophoric dysplasia type 1 was confirmed on autopsy.

**Conclusion:**

This report was aimed to highlight the potential contribution of ultrasound scan in the diagnosis of thanatophoric dysplasia in our setting.

## 1. Background

Skeletal dysplasias are heritable disorders characterized by abnormalities of cartilage and bone. Thanatophoric dysplasia is the most lethal form of skeletal dysplasias. This case is reported because of its rarity and to increase awareness among health professionals, especially sonographers in our local setting.

## 2. Case Presentation

A 33-year-old G3 P2 presented to the facility as a referral on account of an ultrasound scan report suggestive of thanatophoric dysplasia type 1 at 34 weeks. On presentation, the patient complained of lower abdominal pain and epigastric pain. There was no bleeding per vaginum or loss of liquor. She had her booking visit at 4 months, with an ultrasound scan done that showed a single live intrauterine gestation. She had hitherto had five antenatal visits and four scans at the referring clinic. Her pregnancy had been uneventful until this current complaint.

She had been on “Pregnacare” from the third month and had received four doses of sulphadoxine pyrimethamine for malaria prophylaxis. The patient had no history of alcohol use or smoking nor used recreational drugs. She had not taken any herbal preparations during the pregnancy and had no known drug allergies. There was no history of radiation exposure. The patient had no significant personal or family history of congenital anomalies or diabetes.

Her first pregnancy was four years ago and was uneventful. She had spontaneous vaginal delivery at 41weeks. Second pregnancy was two years six months ago. She had a caesarean section done on account of failed induction. Both children are alive and well, with no congenital anomalies.

On examination, the patient was stable with normal vital signs. Her abdomen was grossly enlarged, with epigastric tenderness. Symphysiofundal height was 43 cm, which was larger than the gestational age and the fetal heart rate was 156 bpm.

**Figure 1:**
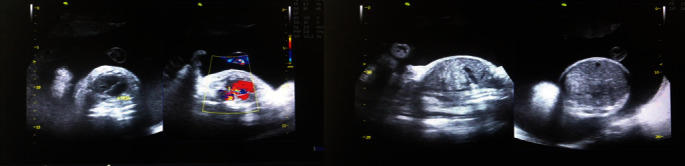
Sonograms showing small thoracic cavity with centrally placed heart, hypoplastic lungs, relatively large abdominal cavity, and increased liquor volume.

The ultrasound scan at 34 weeks showed a biparietal diameter of 37weeks and a femur length of 24 weeks. As shown in Figure 1, the thoracic cage was narrowed with hypoplastic lungs and short ribs, and the heart was centrally occupying the chest. The liquor volume was markedly increased with amniotic fluid index (AFI) of 38.4 cm. The femur, tibia, fibula, humerus, ulna, and radius were shortened (micromelia). Based on these findings, thanatophoric dysplasia type 1 diagnosis was made.

The couple was counselled in detail by the obstetrician, regarding the diagnosis, prognosis, clinical course, and possible complications. The couple decided to terminate the pregnancy and asked for withdrawal of care of the newborn after delivery. A clinical psychologist also gave them counselling. The patient had an elective caesarean section done after a full workup. A female baby weighing 2.25 kg was delivered, with APGARS 2/10 and 1/10 at one and five minutes, respectively. The baby was centrally and peripherally cyanosed. The estimated liquor volume was two litres. The baby was sent to NICU but died within the first hour of life.

**Figure 2:**
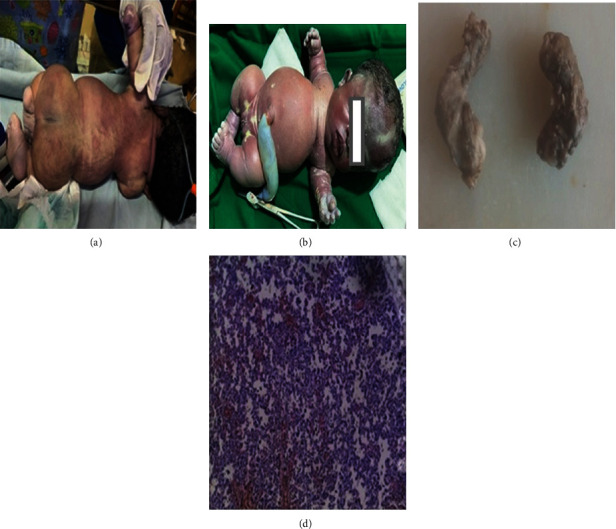
Immediate postdelivery of the female baby at 34 weeks. (a) Showing flat back and spine with dimpling over the sacral region (b) Shows enlarged head (head circumference = 35 cm), small thoracic region (chest circumference (24 cm), and enlarged abdomen (abdominal circumference (30 cm). (c) Bilateral shortening of upper and lower limbs, bowed thighs and legs (femur length (left = 4.5 cm, right = 4.5 cm), foot length (left = 4 cm, right = 4 cm), and short stubby fingers. (d) Section of the lung shows reduced dilated alveolar spaces with thickened congested capillary walls as well as hyperplastic arterioles with duplicated concentric media and narrowing of the lumen; features are suggestive of pulmonary hypoplasia and pulmonary arterial hypertension (medial hypertrophy stage I).

Examination revealed (Figure 2) clinical features of skeletal dwarfism with shortening of both upper and lower limbs. The head was enlarged, with frontal bossing, flattened nasal bridge, short neck, low set ears, coarse and edematous face, macroglossia, and widened anterior fontanel. The spine was flat, with small dimpling over the sacrum and a relatively narrow thorax. Had short stubby fingers, bowed thighs and legs, deep skin creases in all limbs and a protuberant abdomen. Clinical examination findings were consistent with ultrasound features.

**Figure 3:**
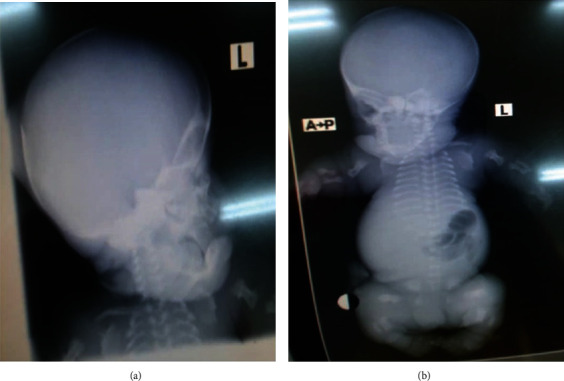
Postmortem radiograph of the skull. (a) Showing macrocephaly, frontal bossing, and flattening nasal bridge. (b) Showing proximal portions of the long limbs are small, giving a rhizomelic appearance. The humeri and femora have typical “telephone handle” bowing with metaphyseal flaring. The iliac bones are hypoplastic. Short horizontal ribs, narrow chest, and small scapulae.

Postmortem showed macrocephaly (head circumference 35 cm), narrow chest cavity with flattened out ribs, hypoplastic lungs, and shortened curved femora (Figure 3). There was no evidence of cardiac, renal, neurologic, or lymphatic abnormalities.

The prenatal ultrasound scan findings, clinical, postmortem, radiologic, and the autopsy findings were consistent with thanatophoric dysplasia type 1.

## 3. Discussion and Conclusion

Thanatophoric dysplasia is a short limb skeletal dysplasia. It is a congenital and sporadic condition. Thanatophoric dysplasia was previously described as thanatophoric dwarfism, but currently, this term is no longer used. Thanatophoros is a Greek word meaning “death bearing.” It was first described by Maroteaux et al. in 1967. It is caused by an autosomal dominant mutation in FGFR 3 gene [1, 2]. Incidence of thanatophoric dysplasia (TD) is 1 : 20,000 to 1 : 50, 000 [2]. This case represents 1 in 750 cases of anomaly scans done within a period of three years at RAAJ Specialist Scan, a private diagnostic centre with branches in the Central and Western Regions of Ghana. Both sexes are equally affected, with no racial or ethnic predisposition [3, 4]. The index case is female.

There are two subtypes. Type 1 accounts for about 80% of cases and type 2 accounts for 20%. The subtypes can be differentiated by the morphologic features of the femur and the shape of the skull. Type 1 is characterized by curved or bowed femurs, and type 2 is characterized by straight femurs and a cloverleaf skull [2, 3, 5]. The current case had TD type 1. Features common to both subtypes include micromelia, short ribs, narrow thorax, brachydactyly, redundant skin folds along the limbs, distinctive facial feature, and relative macrocephaly [2, 5]. All these features were present in the index case. Although formal diagnostic criteria for thanatophoric dysplasia have not been established, diagnosis is based on clinical characteristics and/or radiologic features and/or molecular genetic testing.

Antenatally, skeletal dysplasia can be identified with the use of ultrasonography. Visualizing facial features and soft tissue features such as very short extremities and a small thorax are suggestive. First trimester findings include increased nuchal translucency and shortened long bones [6]. Unfortunately, the earlier scans done at 8 weeks, 14 weeks, and 24 weeks by the referring centre of the index case did not make a diagnosis of thanatophoric dysplasia. Second and third trimester findings include polyhydramnios, narrow chest cavity with short ribs, bowed femur, relative macrocephaly, growth deficiency with limb length below the fifth centile, ventriculomegaly, and brain anomalies [2]. With the exception of the brain anomalies, all the aforementioned features were noted during the third trimester scan at 34 weeks in the index case.

Clinical course usually involves most fetuses dying in utero or some few hours after birth, with respiratory insufficiency usually being the cause of death. This may be due to the narrow chest and hypoplastic lungs or brain stem compression or both [7]. Long-term survivors are rare and need aggressive intervention for complications that may arise [2]. The index case was born deeply cyanosed, with labored breathing and died in the first hour. The autopsy also confirmed the presence of a narrow chest with lung hypoplasia and pulmonary hypertension.

Radiologic features include rhizomelic shortening of long bones, irregular metaphyses of long bones, platyspondyly, bowed femurs, and cloverleaf skull [2]. The index case had a postmortem radiograph showing shortened proximal portions of the long limbs, giving a rhizomelic appearance. The humeri and femora had typical “telephone handle” bowing with metaphyseal flaring. The iliac bones were hypoplastic. Short horizontal ribs, narrow chest, and small scapulae were noted.

Other rarely reported features include cardiac defects, renal, and lymphatic abnormalities. These were absent during the ultrasound scan and autopsy of the index case. Possible pregnancy complications include polyhydramnios, prematurity, malpresentation, and cephalopelvic disproportion from macrocephaly. The index case was complicated with polyhydramnios. Differential diagnoses include osteogenesis imperfecta II, achondrogenesis, achondroplasia, hypophosphatasia, camptomelic dysplasia, and skeletal ciliopathies [2, 3]. Parameters to aid with differential diagnosis include degree of bone mineralization, bowing, presence of fractures, number of digits, and associated anomalies.

The presence of dark blue sclera and fracturing of long bones is characteristic of osteogenesis, distinctive facial features and extreme hypomineralization in achondrogenesis, bowing of only lower limbs with hypoplastic fibula in camptomelic dysplasia, and macrocrania and less prominent shortening of long bones in achondroplasia [1, 5].

Although thanatophoric dysplasia is rare, this case report emphasizes the need for insight regarding the problem and the importance of early prenatal diagnosis to aid in alternative options of termination of pregnancy and to avoid potential pregnancy complications.

## Data Availability

All data generated or analyzed during the study are included in this article.

## Abbreviations

TD: Thanatophoric dysplasia
FGFR 3: Fibroblast growth factor receptor 3
NICU: Neonate intensive care unit
APGARS: Appearance, pulse, grimace, activity, and respiration score
G3P2: Gravidity 3 and parity 2 mother.
